# The Effect of Strength Training Targeting Medial Thigh Muscles on Neuromuscular and Biomechanical Risk Factors for Anterior Cruciate Ligament Injury: A Randomized Controlled Trial

**DOI:** 10.1186/s40798-025-00812-1

**Published:** 2025-01-23

**Authors:** Jiyoung Jeong, Dai-Hyuk Choi, Choongsoo S. Shin

**Affiliations:** 1 Department of Mechanical Engineering, Sogang University, 35 Baekbeom-ro, Mapo-gu, Seoul, 04107 Republic of Korea; 2 Department of Physical Education, Graduate School of Education, Sogang University, 35 Baekbeom-ro, Mapo-gu, Seoul, 04107 Republic of Korea

**Keywords:** Anterior Cruciate Ligament, Injury Prevention, Kinematics, Kinetics, Muscle Activation

## Abstract

**Background:**

Knee valgus loading is thought to be an important contributor to noncontact anterior cruciate ligament (ACL) injuries, but the effects of training programs focusing on decreasing knee valgus loading on lower extremity biomechanics with respect to ACL injury risk remain unclear. Thus, this study aimed to examine the effect of strength training designed to strengthen the medial thigh muscles on lower extremity joint kinematics, kinetics and muscle activity during single-leg landing.

**Methods:**

A total of 35 healthy participants randomly conducted either exercises targeting medial thigh muscles (intervention group) or exercises that did not target specific lower extremity muscles (control group). Three-dimensional hip, knee, and ankle kinematic/kinetic data and muscle activity for lower extremity muscles were obtained during single-leg landing. Two-way analyses of variance were conducted for each dependent variable to determine the effect of 8-week of strength training targeting medial thigh muscles.

**Results:**

The intervention group showed decreased knee varus-valgus excursion (*P* = 0.009), peak valgus moment (*P* = 0.032), and peak hip internal rotation moment (*P* = 0.009) but increased gluteus medius activity in the precontact phase (*P* = 0.012) and vastus medialis-to-vastus lateralis (VM: VL) coactivation ratio in the postcontact phase (*P* = 0.043). The change in coronal plane knee excursion was negatively correlated with both the change in gluteus medius activity (R^2^ = 0.321, *P* = 0.014) and the change in VM: VL coactivation ratio (R^2^ = 0.276, *P* = 0.025).

**Conclusions:**

Strength training targeting medial thigh muscles can modify the biomechanics associated with ACL injuries; thus, this intervention might be considered when designing ACL injury prevention programs to reduce dynamic knee valgus during sports-related tasks.

## Key Points

• Eight weeks of strength training targeting medial thigh muscles alone altered the biomechanical risk factors of anterior cruciate ligament (ACL) injury, whereas the nontargeting strength training did not.

• The coronal plane knee excursion and peak knee valgus moment during single-leg landing decreased resulting from altered motor control strategy after targeted strength training.

• The implementation of this novel strength training targeting the medial quadriceps and hamstrings might be considered when planning ACL injury prevention programs aimed at reducing knee valgus loading.

## Background

Anterior cruciate ligament (ACL) injury is one of the most frequent types of sports injuries of the knee joint. Since ACL injury often leads to long-term consequences, including early onset and/or progression of osteoarthritis [1, 2], preventing ACL injury by investigating the injury mechanisms and risk factors is important. It has been established that multiple factors can contribute to the occurrence of ACL injury [3]. Among them, dynamic knee valgus and peak valgus moment, which are modifiable biomechanical risk factors, are thought to be important contributors to noncontact ACL injuries [4]. The peak valgus moment increased the peak ACL strain, and greater peak ACL strain was shown in participants who landed with valgus alignment than in those with neutral alignment [5]. Thus, the efficacy of intervention training programs focusing on decreasing knee valgus loading needs to be investigated to reduce noncontact ACL injuries.

Medial or lateral muscle groups of the thigh have an influence on the frontal plane loading of the knee joint. A previous study reported that the greater strength of medial thigh muscles (e.g., vastus medialis and semitendinosus/semimembranosus) relative to lateral thigh muscles (e.g., vastus lateralis and biceps femoris) was associated with a lower knee valgus moment during single-leg landing [6]. It also has been documented that the medial muscles of the lower extremity are involved in generating the knee valgus moment to resist the external varus load, while the medial muscles are involved in generating the varus moment in counteracting external valgus load [7–9]. In summary, these results support that an imbalance in the medial-to-lateral quadriceps and hamstrings ratios can contribute to controlling frontal plane knee motion and loading. Previous studies analyzing muscle activation during lower extremity exercises reported that the medial quadriceps/hamstrings muscles can be trained independently by activating the medial thigh muscle groups selectively [10, 11]. Accordingly, strength training targeting the medial quadriceps and hamstrings has been proposed previously [12], but no studies have investigated the effect of this strength training designed to target the medial quadriceps and hamstrings on lower extremity biomechanics with respect to ACL injury risk.

The purpose of this study was to examine the effect of strength training designed to strengthen the medial thigh muscles on lower extremity joint kinematics, kinetics, and muscle activation during single-leg landing. It was hypothesized that medial quadriceps and hamstring trained participants would demonstrate a decreased knee valgus angle and peak valgus moment, as well as an increased medial-to-lateral quadriceps and hamstring activation ratio.

## Methods

### Study Design and Randomization

**Fig. 1:**
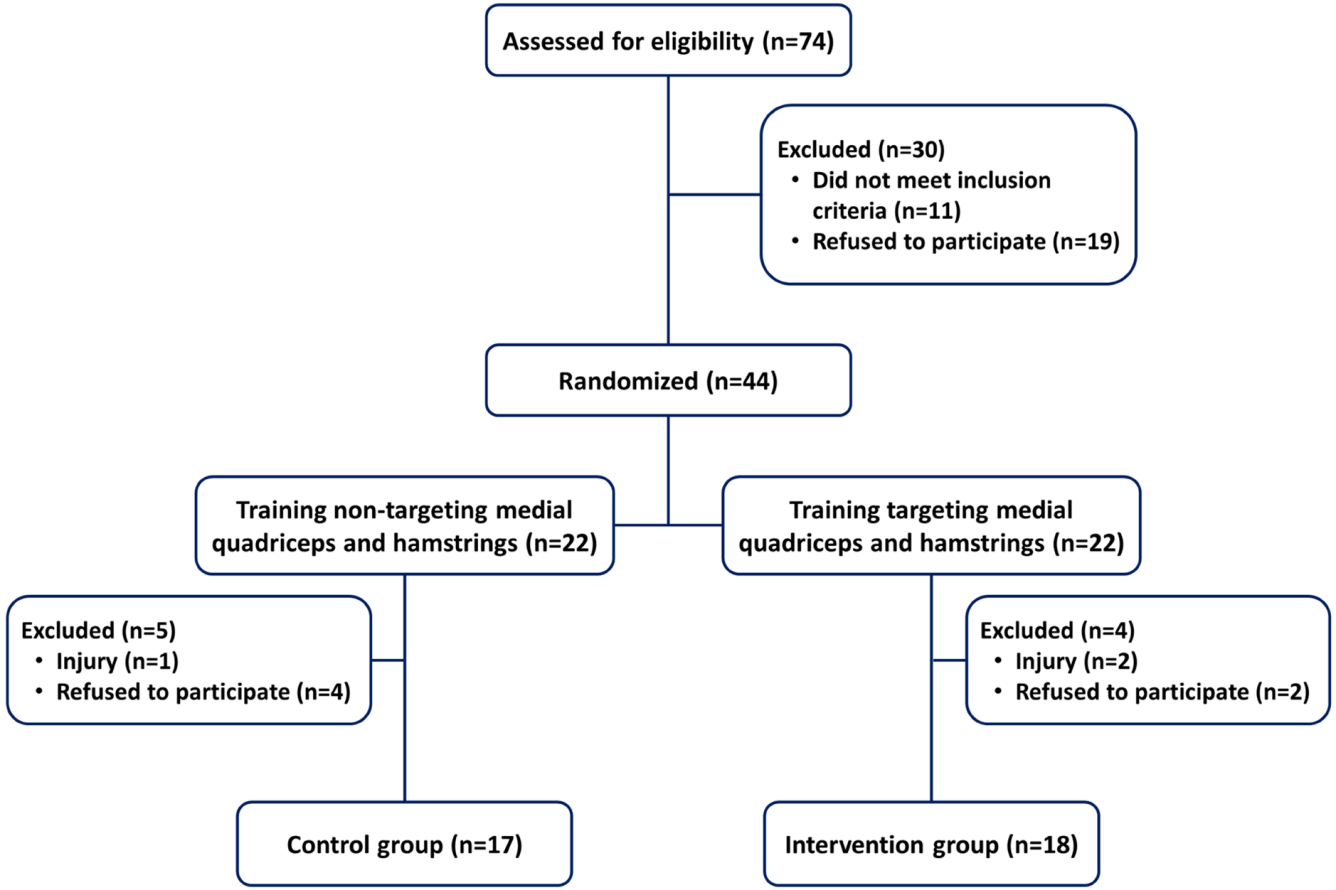
Study flow diagram

This was a randomized controlled study in which the participants were allocated to an intervention group or a control group (Fig. 1). Eligible people who were willing to participate in this study were provided written information about the study, and were required to sign an informed consent form approved by the Institutional Review Board (IRB). Then, participants were randomly assigned to either the intervention group or control group. Randomization was performed using the random number generator in an Excel spreadsheet program, and the investigators were blinded by not informing the participants of their group allocation.

### Participants

A priori power analysis based on the data in the pilot study, which showed an effect size of 0.83 for the medial-to-lateral thigh muscle thickness ratio after strength training targeting the medial quadriceps and hamstrings, was performed using G*power software version 3.1.9.4 (Universitat Kiel, Germany). As a result, the required sample size to obtain a desired power of 0.80 with an alpha level of 0.05 was at least 11 participants.

Participants were eligible for the study if they were recreationally active males and females, defined as participating in some form of physical activity for a minimum of 20 min, 3 times per week, but with no resistance training experience. Participants were excluded if they experienced a lower extremity injury in the past 6 months that prevented participation in physical activity for more than 2 weeks or had a history of ACL injuries or other musculoskeletal injuries requiring surgery.

### Experimental Protocol

Participants conducted exercises targeting medial thigh muscles (intervention group) or exercises that did not target specific lower extremity muscles (control group) for 8 weeks (Table 1). Both strength training programs were developed based on a review of the literature [10, 13–16] and consultation with an expert in athletic training. Participants in both the intervention and training groups were required to complete more than 90% of sessions (at least 22 of the 24 sessions) for inclusion in the final dataset, and the training programs were led by certified sports trainers. The number of sets and repetitions in each set were recorded for each training program. The muscle contraction pace for each repetition was controlled to standardize the rate of contraction across participants. In the first four weeks, participants completed 3 sets of each exercise with a target of 12 repetitions per set when the exercise intensity was 60% of one repetition maximum (1RM), which is the maximum weight that a participant can lift for one repetition. Each repetition was employed for 5 s: contraction for 1 s, holding of the contracted position for 2 s, return to the starting position for 2 s, and then rest for 60 s. In the last four weeks of the study, participants completed 3 sets of each exercise with a target of 20 repetitions per set with 40% of 1RM strength intensity. Each repetition was employed for 1 s, and then there was rest for 20 s. The exercises were conducted with a wide stance and internally rotated foot conditions for the intervention group to strengthen the medial thigh muscles, whereas a normal stance width and neutral foot conditions were applied for the control group. The order of exercises was randomly changed to remove the bias in muscle strengthening, and 1RM was assessed for each exercise prior to strength training.

**Table 1:** Strength training program targeting/nontargeting medial quadriceps and hamstrings for intervention and control group

	Programs	Weeks 1–4	Weeks 5–8
Warm up	1.3 km jogging	15 min	15 min
Strength training programs targeting medial quadriceps/ hamstrings for intervention group	1. Wide leg press 2. Barbell wide squat 3. Leg extension 4. Leg curl with internal rotated foot	• 1 set: - 60% of 1RM - 12 repetitions - For 60 s • Total 3 sets • 60 s rest	• 1 set: - 40% of 1RM - 20 repetitions - For 20 s • Total 5 sets • 20 s rest
	5. Ball squat 6. Plank leg raise with internal rotated foot	• 1 set: - 12 repetitions - For 60 s • Total 3 sets • 60 s rest	• 1 set: - 20 repetitions - For 20 s • Total 5 sets • 20 s rest
	7. Bridge with ball	• 1 set: - Hold for 60 s • Total 3 sets • 60 s rest	• 1 set: - Hold for 30 s with each limb only • Total 3 sets • 60 s rest
Strength training programs nontargeting medial quadriceps/ hamstrings for control group	1. Leg press 2. Barbell squat 3. Leg extension 4. Leg curl with neutral foot position	• 1 set: - 60% of 1RM - 12 repetitions - For 60 s • Total 3 sets • 60 s rest	• 1 set: - 40% of 1RM - 20 repetitions - For 20 s • Total 5 sets • 20 s rest
	5. Squat 6. Plank leg raise with neutral foot position	• 1 set: - 12 repetitions - For 60 s • Total 3 sets • 60 s rest	• 1 set: - 20 repetitions - For 20 s • Total 5 sets • 20 s rest
	7. Bridge without ball	• 1 set: - Hold for 60 s • Total 3 sets • 60 s rest	• 1 set: - Hold for 30 s with each limb only • Total 3 sets • 60 s rest
Stretches	1. Quadriceps 2. Hamstrings 3. Calf muscles 4. Hip muscles	30 s each	30 s each

1RM: one repetition maximum

The concentric isokinetic muscle strength of the quadriceps and hamstrings was measured using an isokinetic dynamometer at 60 °/sec (Biodex Corp., Shirley, NY, USA) before and after both strength training programs. Each participant was positioned in a seated position on the knee testing table with stabilization straps across the trunk and thighs. The dynamometer lever arm was adjusted to the length of the participants’ shank and positioned laterally over the knee joint axis of rotation. Before starting the actual test, explanations of the testing methodology, such as what actions (i.e., knee flexion/extension) were going to be tested and the range of motion, were given. Following three practice repetitions, the highest peak torque value from three maximal concentric isokinetic repetitions was obtained for data collection.

Ultrasound images of the rectus femoris (RF), vastus lateralis (VL), vastus medialis (VM), vastus intermedius (VI), biceps femoris (BF), and semitendinosus (ST) on the dominant limb were obtained using B-mode ultrasound (MicrUs EXT-1 H, Telemed Ultrasound Medical System, Milano, Italy) with a linear array probe (10 MHz). Ultrasound settings, including gain (60 dB), depth (70 mm), and frequency (10 MHz), were set prior to testing. Sufficient ultrasound gel was used to minimize muscle compression of the probe head. With the participant lying supine and both feet fixed to prevent hip external rotation, the thickness of VM was measured at 20% of the distance between the superior tip of the patella and the anterior superior iliac spine, and RF, VI, and VL were measured at 50% of this distance above the patella [17]. All ultrasound images of the BF and ST were scanned in the sagittal plane at 50% of the distance between the greater trochanter and the lateral joint line of the knee in the prone position with the lower limb extended and relaxed [18]. For each session, two ultrasound images were captured, and the mean was calculated for each muscular parameter.

A three-dimensional motion capture system equipped with ten infrared cameras (nine Eagle and one Raptor; Motion Analysis Corp., Santa Rosa, CA, USA) was used to record the motion of the knee joint at a sampling rate of 400 Hz. Reflective markers with the diameter of 12.5 mm were placed on the anatomical bony landmarks: left and right acromion, sternum, right scapula, bilateral anterior superior iliac spines, sacrum, greater trochanter, midpoint of the femur, lateral and medial epicondyles of the femur, lateral and medial plateau of the tibia, midpoint of the tibia, lateral and medial malleolus, calcaneus, and the first and the fifth metatarsal heads. A force plate (9260AA6; Kistler, Winterthur, Switzerland) embedded in the floor was used at a sampling rate of 1200 Hz and was synchronized with the motion capture system to calculate the joint moments. A wireless EMG system (Wave plus wireless, Cometa, Milan, Italy) was used to record muscle activity from the gluteus maximus/medius, RF, VM, VL, ST, BF, and medial/lateral gastrocnemius with a sampling rate of 1200 Hz during single-leg landing. Surface electrodes were attached to the muscle bellies with an inter-electrode distance of 20 mm in recommended locations.

All participants were instructed to perform a single-leg landing by stepping off of a 0.3-m platform without jumping up, using the dominant limb, with folded arms across the chest. Leg dominance was defined as the preferred leg for kicking a ball. The participants were instructed to remain balanced on their dominant leg for at least 2 s after landing. To remove the effect of footwear, all participants wore the same running shoes: Nike Downshifter 8 (NIKE Inc., Beaverton, OR, USA). Prior to the actual experimental trials, each subject in the intervention or control group was given approximately 15 min to walk and run to get used to the provided shoes [19], and each subject was instructed to perform several practice trials to become familiar with the procedures and instrumentation. If a participant jumped up, failed to adopt a stable landing posture by losing balance, failed to keep the foot facing straight ahead or landed with the foot out of the force plate, then the corresponding trials were discarded.

### Data Analysis

All ultrasound images were analyzed using ImageJ software (Version 1.51k, National Institutes of Health, Bethesda, MD) to measure the muscle thickness of the RF, VI, VL, VM, BF, and ST, which was defined as the distance between the superficial and deep fasciae of each muscle. The VM: VL thickness ratio was calculated as the VM thickness divided by the VL thickness, the ST: BF thickness ratio was calculated as the ST thickness divided by the BF thickness, and the medial-to-lateral (M: L) thickness ratio was the sum of the VM and ST thickness divided by the sum of the VL and BF thickness.

Each trial was defined as the period from the initial foot contact to the toe-off, as determined by the force plate recordings. The initial contact of single-leg landing was identified by finding the first frame at which the vertical ground reaction force exceeded 20 N. The measured kinematic and kinetic data were filtered using a zero-lag fourth-order Butterworth low-pass filter at cutoff frequencies of 10 and 20 Hz, respectively. To calculate the joint kinematics, the coordinate systems for each body segment were defined following the methods of a previous study [20]. The joint moments were calculated by solving an inverse dynamics problem using Newton-Euler equations with the segments’ inertial properties based on a previous report [21]. Joint moments were expressed as external moments and normalized to the body weight and height of each participant.

Raw EMG signals were filtered using a digital fourth-order Butterworth high-pass filter with a cutoff frequency of 30 Hz. Following full wave rectification, signals were filtered once again using a digital fourth-order Butterworth filter with a 10 Hz cutoff frequency to obtain the linear envelope of the EMG signals. The processed EMG data were then normalized to the EMG amplitudes obtained from maximal voluntary isometric contractions (MVCs) during manual muscle tests. The mean EMG amplitudes (% MVC) for each muscle during the precontact and loading phases were calculated. The precontact phase was defined as the 100-ms time period prior to initial contact, and the postcontact phase was defined as the 100-ms time period after initial contact [22, 23]. The VM: VL coactivation ratio was calculated as the average EMG amplitude of the VM divided by the average EMG amplitude of the VL, and the ST: BF coactivation ratio was also calculated as the average EMG amplitude of the ST divided by the average EMG amplitude of the BF. In addition, M:L quadriceps and hamstrings coactivation ratio was calculated as the sum of the average EMG amplitudes of the VM and ST divided by the sum of the average EMG amplitudes of the VL and BF. The H: Q coactivation ratio was calculated as the average EMG amplitude of the hamstrings (BF and ST) divided by the average EMG amplitude of the quadriceps (RF, VM, and VL) during the precontact and postcontact phases. A threshold of 10% of the maximum amplitude of the linear envelope representing the muscle burst of interest was used to identify the onset of muscle activity. The changes in the latency between the onset of medial quadriceps activity were quantified by subtracting the onset of VM activity from that of VL.

### Statistical Analysis

The two successful trials were averaged individually and then averaged to generate group mean values and standard deviation. The Kolmogorov–Smirnov test was used to check the normality of the data distribution. The results revealed that all recorded data were normally distributed (*P* > 0.05). A high degree of reliability was demonstrated using a two-way mixed model (intraclass correlation coefficient; ICC_3,1_) to assess the agreement in all muscle thicknesses between the data measured across two trials on separate days by one rater (ICC_3,1_ ≥ 0.961). Although it has already been reported that muscle thickness is used to indirectly predict the force production capacity because it presents a strong positive correlation with maximal muscle strength [24], to confirm the relationship between muscle thickness and the muscle strength of the quadriceps and hamstrings, Pearson’s correlation coefficient was used in this study. Subsequently, Fisher’s r to z test was used to examine group and time as moderators of the significant correlations. A repeated measures 2 × 2 (group × time) analysis of variance was conducted for each dependent muscular and biomechanical variable. Pairwise comparisons were performed to compare each dependent variable between pre- and post-training within each group and between control and intervention groups at each time point, if a significant interaction effect of group-by-time was detected. Changes in kinematic and neuromuscular variables were calculated by subtracting the measured values at pretraining from the measured values at post-training. To determine the relationship between the changes in dependent variables, Pearson’s correlation coefficient was also used. All statistical analyses were performed using SPSS version 23.0 (IBM Corp., Armonk, NY, USA), with a significance level set at 0.05.

## Results

Among the 44 participants, a total of 18 participants (10 males and 8 females) in the intervention group (age: 22.1 ± 1.8 year; height: 167.0 ± 8.2 cm; mass: 59.5 ± 12.0 kg; BMI: 21.2 ± 2.8 kg/m^2^) and 17 participants (8 males and 9 females) in the control group (age: 21.9 ± 1.9 year; height: 169.6 ± 7.5 cm; mass: 64.8 ± 9.2 kg; BMI: 22.5 ± 2.3 kg/m^2^) successfully completed the study and were included in the data analyses. All 9 participants who did not complete the study dropped out because of private reasons such as refusal to participate and injuries (Fig. 1).

**Fig. 2:**
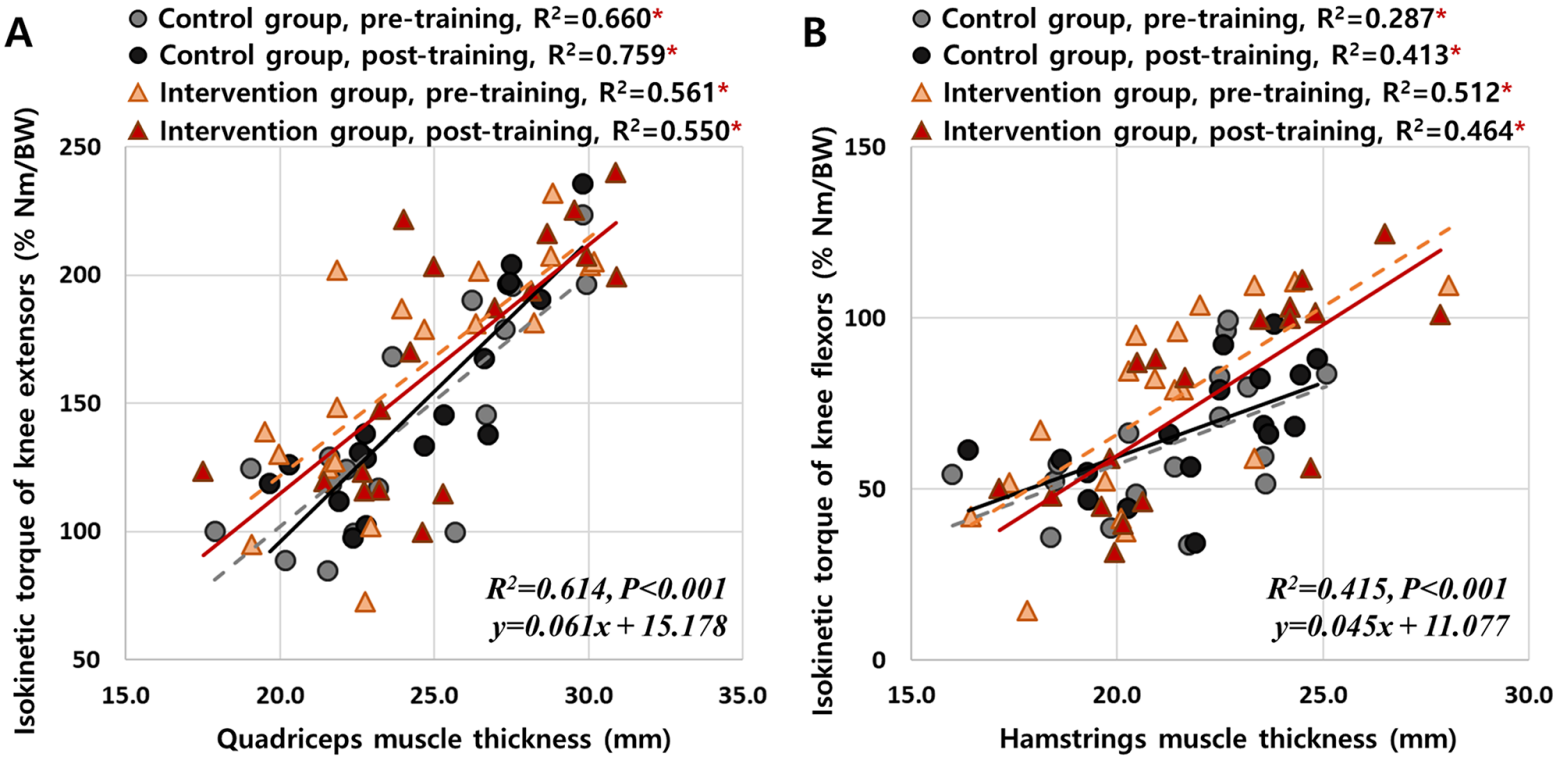
Relationships between the muscle thickness derived from the ultrasound imaging and the isokinetic torque of (**A**) knee extensors and (**B**) flexors. The asterisks (*) indicate significant relationships between muscle thickness and isokinetic torque (*P* < 0.05). BW: body weight

The isokinetic torque of both the knee extensor (R^2^ = 0.614, *P* < 0.001) and flexor (R^2^ = 0.415, *P* < 0.001) were positively correlated with the muscle thickness of the quadriceps (Fig. 2A) and hamstrings (Fig. 2B), respectively. There were no significant group or time differences in the correlations between the isokinetic torque and the muscle thickness of the quadriceps (z = 1.033, *P* = 0.302 and z = 0.279, *P* = 0.780, respectively) and hamstrings (z = 0.789, *P* = 0.430 and z = 0.313, *P* = 0.754, respectively). Thus, muscle thickness can be used as a means of predicting muscle strength in this study.

Significant main effects of time were observed for the muscle thickness of the quadriceps and the hamstrings (*P* < 0.001 for both parameters, Table 2), and significant group × time interactions were observed for VL, VM, and ST thickness. After the strength training targeting the medial quadriceps and hamstrings, the muscle thickness of VM and ST increased by 7.9% and 9.2%, respectively, compared with the corresponding values in pretraining (*P* < 0.001 for both comparisons, Table 2), thereby increasing the medial-to-lateral quadriceps (VM: VL), hamstrings (ST: BF) and thigh muscle ((VM + ST): (VL + BF)) thickness ratio, as well (*P* < 0.001 for all comparisons, Table 2). In addition, the VL and ST thickness were increased by 5.2% and 3.8%, respectively, and the VM: VL thickness ratio was decreased by 4.5% after nontargeted strength training in the control group (*P* = 0.001, *P* = 0.044, and *P* = 0.026, respectively, Table 2). In post-training, the medial-to-lateral thigh muscle thickness ratio was greater by 5.3% in the intervention group than in the control group (*P* = 0.020, Table 2), whereas no differences were found between the control and intervention groups in pretraining.

**Table 2:** Muscle thickness of individual quadriceps and hamstrings in both intervention and control group (mean ± standard deviation)

	Control		Intervention	
	Pre	Post	Pre	Post
Quadriceps (mm)^d^	23.9 ± 3.6	24.7 ± 3.0	24.4 ± 3.7	25.5 ± 3.6
Hamstrings (mm)^d^	21.3 ± 2.4	21.9 ± 2.4	21.0 ± 2.8	22.2 ± 2.9
RF + VI (mm)^d^	35.6 ± 5.7	38.5 ± 6.8	36.2 ± 7.2	38.3 ± 7.0
VL (mm)^a, d^	23.0 ± 2.9	24.2 ± 2.9^b^	24.4 ± 3.8	24.6 ± 3.7
VM (mm)^a, d^	30.2 ± 5.7	30.5 ± 4.7	30.3 ± 4.9	32.7 ± 4.8^b^
BF (mm)	21.5 ± 2.6	22.0 ± 2.6	21.3 ± 3.2	21.8 ± 3.4
ST (mm)^a, d^	21.0 ± 2.4	21.8 ± 2.2^b^	20.6 ± 2.7	22.5 ± 2.8^b^
VM: VL thickness ratio^a, d^	1.32 ± 0.19	1.26 ± 0.15^b^	1.25 ± 0.14	1.33 ± 0.13^b^
ST: BF thickness ratio^a^	0.98 ± 0.07	0.99 ± 0.04	0.97 ± 0.09	1.04 ± 0.09^b^
M: L thickness ratio^a, d^	1.15 ± 0.09	1.13 ± 0.07	1.12 ± 0.07	1.19 ± 0.07^b, c^

^a^Significant interaction effects (*P* < 0.05); ^b^Significant differences between pre- and post-training in pairwise comparisons for each group of control and intervention (*P* < 0.05); ^c^Significant differences between control and intervention groups after training in pairwise comparisons (*P* < 0.05); ^d^Significant time effects (*P* < 0.05). RF: rectus femoris; VI: vastus intermedius; VL: vastus lateralis; VM: vastus medialis; BF: biceps femoris; ST: semitendinosus; M: medial; L: lateral

Significant group × time interaction effects were observed for knee varus-valgus excursion, peak knee valgus moment, and peak hip internal rotation moment (*P* = 0.036, *P* = 0.042, and *P* = 0.037, respectively, Table 3). Pairwise comparisons showed that there were decreases in the varus-valgus excursion, the peak knee valgus, and the peak hip internal rotation moments during single-leg landing in the intervention group (*P* = 0.009, *P* = 0.032, and *P* = 0.009, respectively), while no differences were found in the control group (Table 3). No significant differences were found in all kinematic and kinetic variables between the control and intervention groups in pretraining, but the varus-valgus excursion in post-training was lower in the intervention group than in the control group (*P* = 0.013, Table 3). The peak knee valgus moment was positively correlated with the peak hip internal rotation moment (R^2^ = 0.212, *P* = 0.005), and the peak knee valgus moment (91 ± 22 ms) occurred before the time point of the peak hip internal rotation moment (128 ± 16 ms) in the intervention group (*P* < 0.001).

**Table 3:** The 3D kinematics and kinetics of hip, knee, and ankle joint in both intervention group (i.e., targeted strength training group) and control group (i.e., nontargeted strength training group) during single-leg landing (mean ± standard deviation)

	Control			Intervention	
	Pre		Post	Pre	Post
***Joint angles (degree)***					
Hip flexion at IC		24.0 ± 5.0	24.3 ± 6.3	22.8 ± 5.4	20.3 ± 5.3
Hip abduction at IC		4.6 ± 3.1	4.0 ± 2.6	3.3 ± 4.6	3.8 ± 3.3
Hip external rotation at IC		13.2 ± 7.3	15.0 ± 6.6	15.9 ± 7.2	16.9 ± 7.9
Peak hip flexion		36.5 ± 5.7	36.7 ± 8.1	35.4 ± 7.8	32.5 ± 7.3
Peak hip abduction		7.6 ± 3.6	7.0 ± 3.0	7.0 ± 3.0	6.4 ± 3.9
Peak hip adduction		4.4 ± 3.6	5.0 ± 3.9	5.4 ± 4.7	4.6 ± 4.1
Peak hip internal rotation		3.7 ± 5.9	3.2 ± 5.7	1.1 ± 6.5	0.9 ± 7.9
Abduction-adduction excursion		12.0 ± 1.8	12.1 ± 2.2	11.0 ± 2.5	10.9 ± 1.9
Knee flexion at IC		21.0 ± 4.0	20.0 ± 4.2	19.3 ± 2.9	17.9 ± 3.5
Knee valgus at IC		1.7 ± 1.4	1.8 ± 1.3	1.3 ± 1.1	1.3 ± 1.2
Tibial internal rotation at IC		1.3 ± 1.9	0.9 ± 1.7	1.6 ± 2.3	1.3 ± 1.2
Peak knee flexion		59.6 ± 5.4	58.5 ± 6.9	58.5 ± 4.9	57.8 ± 4.3
Peak knee valgus		2.2 ± 1.5	2.7 ± 1.7	1.9 ± 1.6	1.7 ± 1.2
Peak tibial internal rotation		4.5 ± 2.3	3.8 ± 2.1	4.7 ± 2.2	4.1 ± 2.2
Varus-valgus excursion^a^, ^d^		7.4 ± 2.3	7.5 ± 2.3	6.5 ± 1.5	5.8 ± 1.3^b^, ^c^
Ankle plantarflexion at IC		29.1 ± 8.3	30.5 ± 10.2	32.9 ± 6.8	34.0 ± 5.0
Ankle inversion at IC		2.9 ± 7.4	3.2 ± 5.3	4.7 ± 3.9	4.7 ± 4.0
Ankle internal rotation at IC		1.4 ± 4.9	2.2 ± 6.5	-0.3 ± 5.6	1.2 ± 4.9
Peak ankle dorsiflexion		17.2 ± 4.4	16.9 ± 5.3	16.8 ± 3.7	16.9 ± 3.1
Peak ankle eversion		6.0 ± 5.2	5.5 ± 3.5	4.4 ± 2.9	4.0 ± 3.3
Peak ankle external rotation		5.8 ± 4.9	6.3 ± 6.0	7.2 ± 5.5	6.0 ± 4.5
Inversion-eversion excursion		10.7 ± 4.9	10.2 ± 4.3	10.5 ± 3.8	9.6 ± 4.6
Joint moments (Nm/(BW*Ht))					
Peak hip flexion^d^		0.158 ± 0.043	0.155 ± 0.063	0.127 ± 0.035	0.112 ± 0.045
Peak hip abduction		0.197 ± 0.031	0.201 ± 0.030	0.213 ± 0.026	0.213 ± 0.027
Peak hip internal rotation^a^		0.067 ± 0.011	0.068 ± 0.014	0.072 ± 0.012	0.068 ± 0.009^b^
Peak knee flexion		0.162 ± 0.030	0.164 ± 0.032	0.248 ± 0.258	0.181 ± 0.015
Peak knee valgus^a^		0.095 ± 0.020	0.098 ± 0.020	0.100 ± 0.017	0.091 ± 0.014^b^
Peak tibial internal rotation		0.023 ± 0.010	0.021 ± 0.008	0.039 ± 0.070	0.021 ± 0.004
Peak ankle plantarflexion		0.164 ± 0.035	0.150 ± 0.037	0.157 ± 0.020	0.151 ± 0.018
Peak ankle inversion		0.026 ± 0.046	0.012 ± 0.011	0.011 ± 0.013	0.011 ± 0.009
Peak internal rotation		0.010 ± 0.004	0.010 ± 0.005	0.007 ± 0.005	0.009 ± 0.004

^a^Significant interaction effects (*P* < 0.05); ^b^Significant differences between pre- and post-training in pairwise comparisons for each group of control and intervention (*P* < 0.05); ^c^Significant differences between control and intervention groups after training in pairwise comparisons (*P* < 0.05); ^d^Significant group effects (*P* < 0.05). IC: initial contact; BW: body weight; Ht: height

**Fig. 3:**
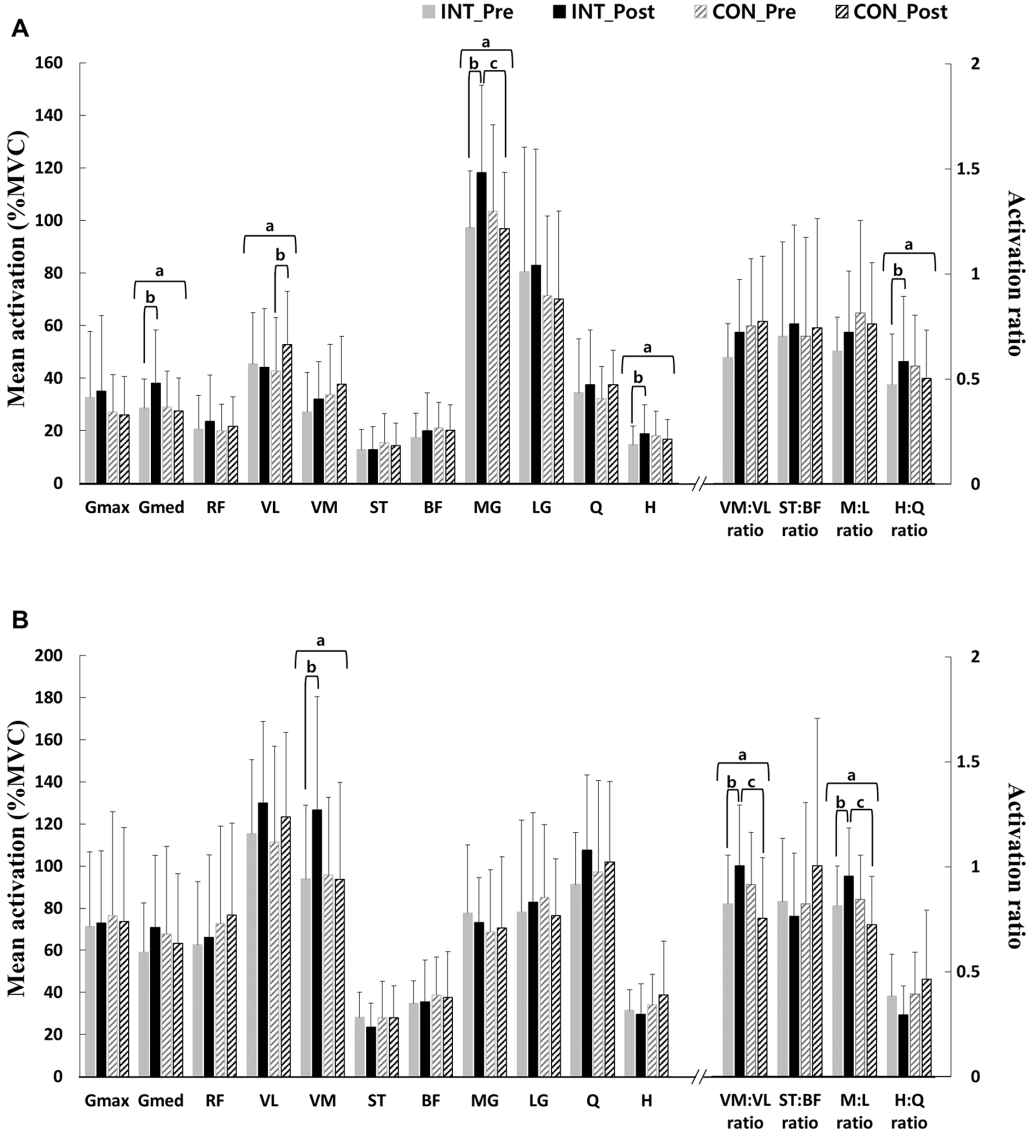
Mean muscle activation amplitude (% MVC) and SD during (**A**) precontact phase and (**B**) postcontact phase for both intervention (targeted strength training) and control group (nontargeted strength training). ^a^Significant interaction effects (*P* < 0.05); ^b^Significant differences between pre- and post-training in pairwise comparisons for each group of control and intervention (*P* < 0.05); ^c^Significant differences between control and intervention groups after training in pairwise comparisons (*P* < 0.05). MVC: maximal voluntary isometric contractions; INT: intervention; CON: control; Gmax: gluteus maximus; Gmed: gluteus medius; RF: rectus femoris; VL: vastus lateralis; VM: vastus medialis; ST: semitendinosus; BF: biceps femoris; MG: medial gastrocnemius; LG: lateral gastrocnemius; M: medial; L: lateral; Q: quadriceps; H: hamstrings

Significant group × time interaction effects for the muscle activity amplitudes of the gluteus medius, VL, and medial gastrocnemius were observed, as well as for the hamstrings and H: Q coactivation ratio in the precontact phase (*P* < 0.05 for all parameters, Fig. 3A). In the postcontact phase, significant group × time interaction effects were also observed for the muscle activity amplitudes of VM and the VM: VL and M: L coactivation ratios (*P* < 0.05 for all parameters, Fig. 3B). Pairwise comparisons showed that there were increases in muscle activity in the precontact phase of the gluteus medius (*P* = 0.012), medial gastrocnemius (*P* = 0.011), hamstrings (*P* = 0.020), and H: Q coactivation ratio (*P* = 0.033) in the intervention group, while there was an increase in VL activity in the precontact phase (*P* = 0.015) in the control group (Fig. 3). The medial gastrocnemius activity in the precontact phase after training was significantly greater in the intervention group than in the control group (*P* = 0.032, Fig. 3). In addition, pairwise comparisons also showed that there were significant increases in muscle activity in the postcontact phase of VM (*P* = 0.004), the VM: VL coactivation ratio (*P* = 0.043), and the M: L coactivation ratio (*P* = 0.036) in the intervention group. Both VM: VL and the M: L coactivation ratio in the post-contact phase were greater in the intervention group than in the control group after strength training (*P* = 0.015 and *P* = 0.007, respectively). There were no significant differences in muscle activity amplitudes between the control and intervention groups in pretraining. The onset timing difference between VL and VM before initial contact during single-leg landing was altered after strength training in the intervention group (pre: 12.9 ± 31.7 ms, post: -8.5 ± 23.2 ms, *P* = 0.007). That is, the onset of VM preceded that of VL after targeted strength training.

The change in coronal plane knee excursion was negatively correlated with both the change in gluteus medius activity in the precontract phase (R^2^ = 0.321, *P* = 0.014) and the change in the VM: VL coactivation ratio in the postcontact phase (R^2^ = 0.276, *P* = 0.025) in the intervention.

## Discussion

The main finding of this study was that 8 weeks of targeted strength training reduced the peak knee valgus moment, peak hip internal rotation moment, and dynamic coronal plane excursion and increased gluteus medius activity and the VM: VL coactivation ratio during single-leg landing, whereas nontargeted strength training did not. These results demonstrate the effects of novel strength training targeting the medial quadriceps and hamstrings on knee and hip biomechanics as a single intervention, thereby supporting the significance of strength training with the specific purpose of reducing ACL injury risk factors in prevention programs.

Varus-valgus excursion significantly decreased after targeted strength training in this study. According to a previous prospective study, athletes who showed greater coronal plane knee angles at the time point of both initial contact and peak value sustained a higher number of ACL injuries than those who had lesser angles [4]. Another prospective study on a group of individuals with ACL reconstruction also reported that athletes with an increase in total coronal plane knee movement were over 3 times more likely to incur a reinjury than those with reduced coronal plane motion [25]. In addition, females showed greater coronal plane excursion during landing than males [26, 27], which is in accordance with the greater incidence of ACL injury in females. Given that varus-valgus excursion seemed to be an important measure as a predictor of ACL injury, strength training targeting medial thigh muscles might be beneficial for the risk of noncontact ACL injuries by reducing the coronal plane oscillation of the knee joint during landing.

**Fig. 4:**
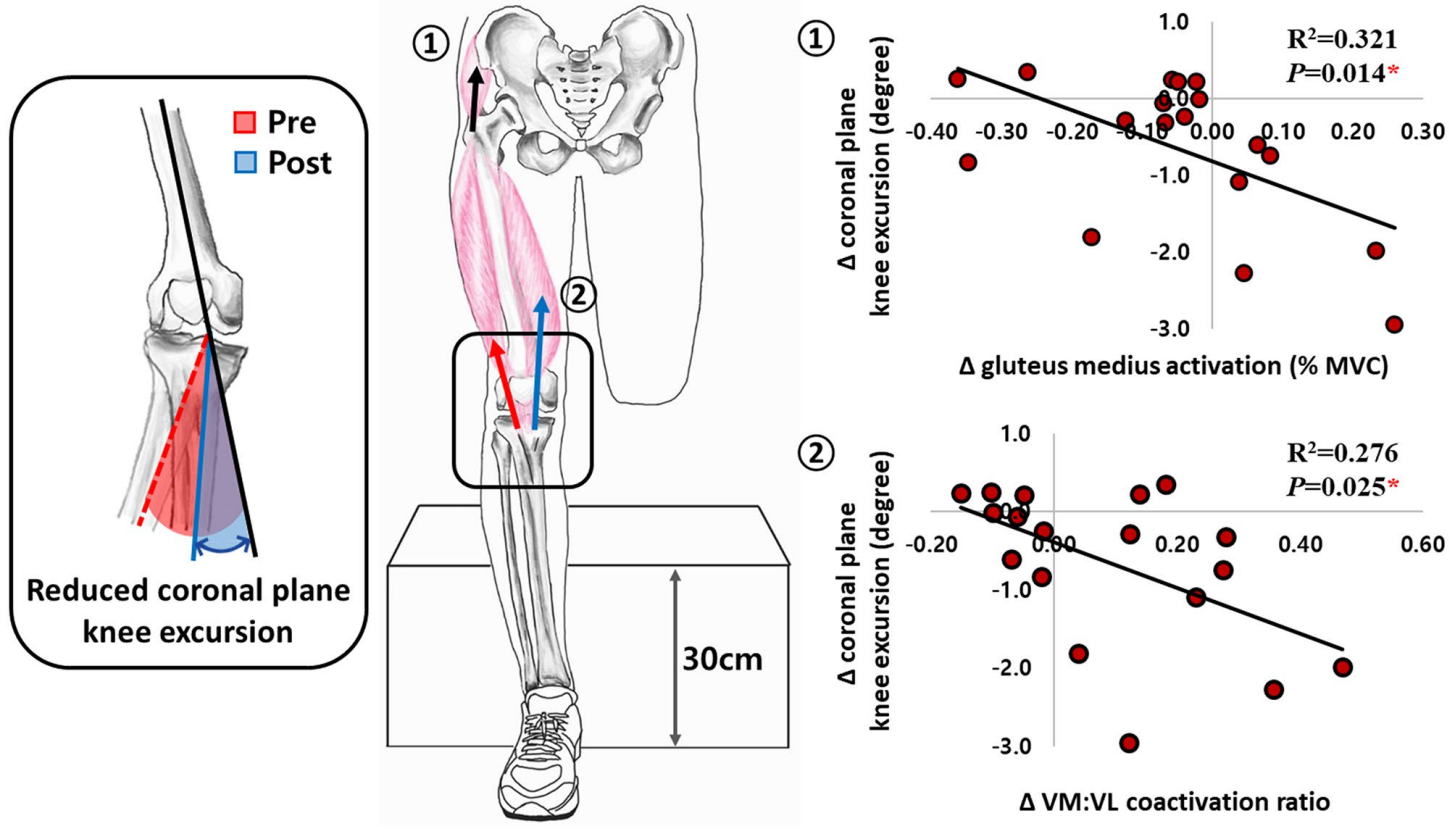
Relationship between the changes in knee varus-valgus excursion with the changes in gluteus medius activation in precontact phase and the changes in vastus medialis to vastus lateralis (VM: VL) coactivation ratio in postcontact phase during single-leg landing after strength training targeting medial quadriceps and hamstrings. The asterisks (*) indicate significant relationships (*P* < 0.05). MVIC: maximal voluntary isometric contractions

Altered muscle activity of the lower extremity after strength training targeting the medial quadriceps and hamstrings likely decreases knee joint coronal plane excursion. The gluteus medius activity in the precontact phase and the VM: VL coactivation ratio in the postcontact phase increased after targeted strength training, and the changes in both altered activities were negatively correlated with the changes in the coronal plane excursion (Fig. 4). These results indicate that the higher the ratio of M: L quadriceps and gluteus medius activity, the smaller the varus-valgus excursion during single-leg landing after targeted strength training. It has been previously reported that the increased VM: VL coactivation ratio after core muscle strength training was negatively correlated with the knee valgus angle during the cutting task [28]. In addition, hip adduction is the primary contributor to excessive dynamic knee valgus by causing the knee joint to move medially [29]. Thus, the decreased varus-valgus excursion during single-leg landing appears to be the consequence of an altered motor control strategy after strength training targeting the medial thigh muscles.

Strength training targeting the medial quadriceps and hamstrings seemed to be beneficial with respect to knee injuries by changing the motor control of lower extremity muscles. Our results show that the onset timing of VL occurred before VM at baseline, but the onset of VM preceded that of VL after targeted strength training. In addition, gluteus medius activation and the H: Q coactivation ratio in the precontact phase increased in the intervention group. It has been established that preparatory activation strategies can protect the joint from excessive load by contributing to joint stabilization during dynamic tasks [30, 31]. A previous case study also reported that athletes with delayed onset timing after initial contact during landing sustained ACL injury [32]. Moreover, based on the evidence that demonstrates a difference in the onset timing of VM and VL in people with patellofemoral pain syndrome, the efficacy of physical therapy programs for patellofemoral pain syndrome, which can alter the VM onset timing relative to VL, has been shown, with a positive clinical outcome [33]. It has also been reported that the increased anticipatory hip abductor (i.e., gluteus medius) contraction can decrease the risk of ACL injury during landing [34], and less hamstring activation with greater quadriceps activation during side-step cutting can cause ACL injury [35]. Taken together, these results show that this novel strength training program that targets medial thigh muscles could be used in various injury prevention programs, including patellofemoral pain syndrome or ACL injury, by changing the neuromuscular control strategy.

Our results also showed significantly reduced peak knee valgus moment and peak hip internal rotation moment after targeted strength training. According to previous cadaveric studies, the peak knee valgus moment has been implicated as a major mechanism of ACL injury occurring during simulated landing by inducing high ACL strains [36–38]. Since the knee valgus moments predicted ACL injury risk with 73% sensitivity and 78% specificity [4], participants in the intervention group might be at lower risk of sustaining an ACL injury after targeted strength training. Adjacent joints such as the hip and ankle may contribute to ACL injury, as the knee is a part of the kinetic chain [3]. However, since the peak knee valgus moment occurred before the peak internal rotation moment was shown during single-leg landing and there was a positive correlation between those parameters in this study, the knee valgus moment might lead to the hip internal rotation moment as a kinetic chain from the knee toward the hip joint. The force can be conveyed from the floor to the knee through the foot, and thus, the foot position also significantly affects the knee joint kinematics and kinetics [39]; no differences, however, were found in ankle kinematics and kinetics after targeted strength training in this study. All things considered, the strengthened medial quadriceps and hamstrings decreased the peak knee valgus moment, which is a powerful risk factor for ACL injury, thereby also decreasing the peak hip internal rotation moment during single-leg landing.

No significant differences in kinematics and kinetics during single-leg landing were found after nontargeted strength training in the control group in this study. A few studies investigating the effect of lower extremity muscle strengthening on knee joint biomechanics reported that lower extremity strength training alone did not significantly alter knee joint kinematics and kinetics during the dynamic task [40, 41], and these results are in accordance with our findings. Considering that targeted strength training reduced the dynamic coronal plane knee excursion and peak knee valgus moment during landing, which are known predictors of ACL injury, strength training targeting the medial quadriceps and hamstrings seemed to be sufficient to reduce the risk of noncontact ACL injury as a single intervention.

Both the quadriceps and hamstrings were strengthened over time regardless of training type. However, when examining the individual muscle strength of the quadriceps and hamstrings in detail, different proportions of muscle strength were found between the groups. The muscle thickness of the VM and ST increased; therefore, the VM: VL, ST: BF, and M: L thickness ratios increased after targeted strength training in the intervention group. In contrast, the VM: VL thickness ratio decreased after nontargeted strength training in the control group. Therefore, the proportion of strengthened muscle can be modified depending on the specific purpose of the strength training.

There are some limitations to this study. First, the actual ACL injury rate in participants after the completion of training was not tracked in this study. Future studies on the effect of strength training targeting the medial quadriceps and hamstrings on the incidence of ACL injuries are needed to evaluate whether the observed changes in biomechanical parameters from the strengthened medial thigh muscles actually resulted in decreased ACL injury rates. In addition, individual muscle thicknesses were only examined for the quadriceps and hamstrings, and other lower extremity muscles were not measured in this study. Muscle strength of non-knee-spanning muscles such as the gluteal muscle can also be increased after training, thereby contributing to lower extremity joint kinematics and kinetics. Thus, additional measurements of other muscle thicknesses in the lower extremity are recommended to gain a deeper understanding of the effect of targeted strength training on ACL injury risk factors. Furthermore, the muscle thickness at a single site was measured in this study, so it seemed difficult to fully reflect the muscle volume, which is directly associated with muscle strength. Quantification of muscle volume using MRI is recognized as the gold standard for providing accurate estimations of muscle size, but with relatively simple and less expensive measurements, measurement of muscle thickness using ultrasound can be more useful as a means of predicting individual muscle strength because there were significant positive correlations between muscle thickness and the isokinetic muscle strength of the quadriceps and hamstrings in this study. Finally, sex differences in adaptation to strength training were not considered in this study. Further studies to determine if there are sex-based differences in the effect of strength training targeting medial thigh muscles on lower extremity joint biomechanics are needed to provide comprehensive insight into the targeted strength training effects on ACL injury risk.

## Conclusion

This study provides evidence that targeted strength training alone alters the biomechanical risk factors for ACL injury by reducing coronal plane knee excursion, peak knee valgus, and peak hip internal rotation moments during single-leg landing. Thus, the current study results suggest that the implementation of strength training targeting the medial quadriceps and hamstrings might be considered when planning ACL injury prevention programs aimed at reducing dynamic knee valgus occurring in single-leg landing.

## Data Availability

The datasets used and/or analyzed during the current study are available from the corresponding author on reasonable request.

## Abbreviations

1RM: 1 repetition maximum
ACL: Anterior cruciate ligament
BF: Biceps femoris
EMG: Electromyography
M: L: Medial-to-lateral
RF: Rectus femoris
ST: Semitendinosus
VI: Vastus intermedius
VL: Vastus lateralis
VM: Vastus medialis
